# Sodium-glucose cotransporter 2 inhibitor versus metformin as first-line therapy in patients with type 2 diabetes mellitus: a multi-institution database study

**DOI:** 10.1186/s12933-020-01169-3

**Published:** 2020-11-09

**Authors:** Tien-Hsing Chen, Yan-Rong Li, Shao-Wei Chen, Yu-Sheng Lin, Chi-Chin Sun, Dong-Yi Chen, Chun-Tai Mao, Michael Wu, Chih-Hsiang Chang, Pao-Hsien Chu, Victor Chien-Chia Wu

**Affiliations:** 1 Department of Cardiology, Keelung Chang Gung Memorial Hospital, Keelung, Taiwan; 2 Division of Endocrinology and Metabolism, Department of Internal Medicine, Linkou Chang Gung Memorial Hospital, Taoyuan, Taiwan; 3 Department of Cardiothoracic and Vascular Surgery, Linkou Chang Gung Memorial Hospital, Taoyuan, Taiwan; 4 Department of Cardiology, Chiayi Chang Gung Memorial Hospital, Chiayi, Taiwan; 5 Department of Ophthalmology, Keelung Chang Gung Memorial Hospital, Keelung, Taiwan; 6 Division of Cardiology, Linkou Medical Center, Linkou Chang Gung Memorial Hospital, No. 5, Fuxing Street, Guishan District, Taoyuan, 33305 Taiwan; 7 Divison of Cardiovascular Medicine, Rhode Island Hospital, Warren Alpert School of Medicine, Brown University, Providence, USA; 8 Department of Nephrology, Kidney Research Center, Linkou Chang Gung Memorial Hospital, Taoyuan, Taiwan

**Keywords:** Type 2 diabetes mellitus, Sodium-glucose co-transporter 2 inhibitor, Metformin, Cardiovascular outcome

## Abstract

**Background:**

Sodium-glucose co-transporter 2 inhibitors (SGLT2i) has shown evidence of cardiovascular benefit in patients with type 2 diabetes mellitus (T2DM). Currently metformin is the guideline-recommended first-line treatment. We aimed to investigate the benefit of SGLT2i vs metformin as first-line therapy.

**Methods:**

Electronic medical records from Chang Gung Research Database during 2016–2019 were retrieved for patients with T2DM. Patients aged < 20, not receiving anti-diabetic medication, first-line treatment neither metformin nor SGLT2i were excluded. Primary outcomes were heart failure hospitalization, acute coronary syndrome, ischemic stroke, and all-cause mortality. Patients were followed up for events or December 31, 2019, whichever comes first.

**Results:**

After exclusion criteria, a total of 41,020 patients with T2DM were eligible for analysis. There were 1100 patients with SGLT2i as first-line and 39,920 patients with metformin as first-line treatment. IPTW was used for propensity score matching. During one year follow-up, the hazard ratio (HR) of patients on SGLT2i as first-line treatment to patients on metformin as first-line treatment were HR 0.47 (95% CI 0.41–0.54, p < 0.0001) in heart failure hospitalization, HR 0.50 (95% CI 0.41–0.61, p < 0.0001) in acute coronary syndrome, HR 1.21 (95% CI 1.10–1.32, p < 0.0001) in ischemic stroke, and HR 0.49 (95% CI 0.44–0.55, p < 0.0001) in all-cause mortality.

**Conclusions:**

In patients with T2DM, SGLT2i as first-line treatment may be associated with decreased events of heart failure hospitalization, acute coronary syndrome, and all-cause mortality, compared with metformin as first-line treatment. However, there may be an increased events of ischemic stroke using SGLT2i compared to metformin.

## Introduction

Cardiovascular events associated with diabetes mellitus are well-documented complications of diabetes disease progression, especially for type 2 diabetes mellitus (T2DM), which is considered as the coronary heart disease equivalent [1, 2]. Newly introduced sodium-glucose co-transporter-2 inhibitors (SGLT2i) reduce serum glucose load with mechanisms completely different from previous anti-diabetic medication categories [3]. SGLT2i such as empagliflozin, dapagliflozin, and canagliflozin were recently studied in several large randomized controlled cardiovascular (CV) outcomes trials EMPAG-REG, DECLARE-TIMI 58, CANVAS [4–6], showing that SGLT2i reduced major cardiovascular events (MACE), improved heart failure, and had greater benefits in patients with established atherosclerotic cardiovascular disease. Meta-analysis also noted SGLT2i protect against CV disease and death in diverse subsets of patients with T2DM regardless of CV disease history [7]. In the lately published DAPA-HF trial, patients with HFrEF irrespective of diabetes status were randomized to dapagliflozin versus placebo, and dapagliflozin was associated reduced cardiovascular death, hospitalization for heart failure, or urgent heart failure visit regardless of diabetes status [8].

Together, these landmark trials have helped encouraged diabetologists and cardiologists to revise and upgrade role of SGLT2i to second-line therapy after metformin or first-line if there is the compelling evidence. Guidelines published by American Diabetes Association (ADA) and American Association of Clinical Endocrinology have made updates to reflect this change in recommendations [9–11]. In 2019 the guideline by European Society of Cardiology and European Association for the Study of Diabetes, SGLT2i was put forth as the first-line treatment recommendation if the patients have atherosclerotic cardiovascular disease (ASCVD), or high/very high CV risk [12]. However, the 2020 ADA guidelines remain still committed to metformin as the first-line therapy for T2DM patients but can go ahead to use SGLT2i if T2DM patients have ASCVD, chronic kidney disease, or heart failure [13]. Therefore in this study, we aimed to investigate the CV outcomes of T2DM patients that are prescribed with either SGLT2i or metformin as the first-line treatment.

## Methods

### Data source

In this retrospective cohort study, patient data were obtained from the largest health-care provider in Taiwan, Chang Gung Memorial Hospital System, comprising three major teaching hospitals and four tertiary-care medical centers [14–17]. The hospital identification number of each patient was encrypted and de-identified to protect their privacy. Therefore, informed consent was waived for this study. The diagnosis and laboratory data could be linked and continuously monitored using consistent data encryption. The institutional review board of Chang Gung Memorial Hospital approved the study protocol.

### Study patients

By searching electronic medical records from the Chang Gung Research Database (CGRD) between January 1, 2016 and December 31, 2019, we retrieved patients with diagnosis of T2DM. We excluded patients age < 20 years old, not received anti-diabetic medication, and first-line treatment neither SGLT2i nor metformin. We then separated patients into first-line anti-diabetic medication that were either SGLT2i or metformin. The prescriptions can be added with other group of anti-diabetic medication in the 3-month follow-up clinic visit if HbA1c was not at goal. These patients were followed up for events or till December 31, 2019, whichever comes first.

### Covariate and study outcomes

Disease was detected using International Classification of Diseases, 9th Revision, Clinical Modification (ICD-9-CM) codes. Covariates included age, sex, diabetes duration, comorbidity, medications, laboratory values, and follow-up years (Table 1). The comorbidity was defined as having two outpatient diagnoses or one discharge diagnosis. Most diagnostic codes of these comorbidities have been validated in previous national database studies. Usage of medication was retrieved based on claim data in the previous year.

**Table 1:** Clinical characteristics of study population before and after propensity score matching

Variable	Before			IPTW		
	SGLT2i (*n* = 1100)	Metformin (*n* = 39,920)	p value	SGLT2i (*n* = 1100)	Metformin (*n* = 39,920)	ASMD
Age, years	57.6 ± 13.0	59.3 ± 12.9	< 0.001	61.2 ± 86.8	59.4 ± 13.1	0.03
Male	697 (63.36%)	22,368 (56.03%)	< 0.001	53.17%	56.21%	0.06
Diabetes duration, year	1.42 ± 3.50	1.12 ± 2.94	< 0.001	1.4 ± 21	1.1 ± 3	0.02
Comorbidity						
Hypertension	735 (66.82%)	23,983 (60.08%)	< 0.001	66.09%	60.86%	0.11
Hyperlipidemia	607 (55.18%)	21,410 (53.63%)	0.31	49.40%	53.94%	0.09
Coronary artery disease	304 (27.64%)	6745 (16.90%)	< 0.001	19.08%	17.45%	0.04
Myocardial infarction	102 (9.27%)	1527 (3.83%)	< 0.001	5.01%	4.07%	0.05
Ischemic stroke	82 (7.45%)	5943 (14.89%)	< 0.001	18.82%	15.10%	0.10
Peripheral artery disease	35 (3.18%)	932 (2.33%)	0.068	3.34%	2.43%	0.05
Heart failure	113 (10.27%)	2343 (5.87%)	< 0.001	5.22%	6.16%	0.04
Atrial fibrillation	66 (6.00%)	1364 (3.42%)	< 0.001	7.17%	3.58%	0.16
Chronic kidney disease	222 (20.18%)	6303 (15.79%)	< 0.001	15.04%	16.05%	0.03
Malignancy	102 (9.27%)	4863 (12.18%)	0.004	9.82%	12.50%	0.09
Medication						
ACEI or ARB	713 (64.82%)	21,486 (53.82%)	< 0.001	60.00%	55.04%	0.10
ARNI	34 (3.09%)	199 (0.50%)	< 0.001	0.44%	0.59%	0.02
Alpha-blockers	66 (6.00%)	1992 (4.99%)	0.13	4.88%	5.17%	0.01
Beta-blockers	593 (53.91%)	16,858 (42.23%)	< 0.001	47.03%	43.49%	0.07
Dihydropyridine CCB	365 (33.18%)	16,042 (40.19%)	< 0.001	46.98%	40.97%	0.12
Non-dihydropyridine CCB	91 (8.27%)	3113 (7.8%)	0.563	9.21%	8.03%	0.04
Digoxin	26 (2.36%)	755 (1.89%)	0.258	1.93%	1.97%	0.00
Ivabradine	27 (2.45%)	171 (0.43%)	< 0.001	0.49%	0.51%	0.00
Nitrates	314 (28.55%)	7168 (17.96%)	< 0.001	16.75%	18.66%	0.05
Diuretics	295 (26.82%)	10,348 (25.92%)	0.504	33.20%	26.69%	0.14
Antiplatelet	429 (39.00%)	13,042 (32.67%)	< 0.001	39.83%	33.56%	0.13
Anticoagulant	76 (6.91%)	1937 (4.85%)	0.002	8.16%	5.07%	0.12
Statin	709 (64.45%)	23,327 (58.43%)	< 0.001	64.57%	59.51%	0.10
Glucose lowering agents						
Metformin	0 (0%)	39,920 (100%)	< 0.001	46.17%	100%	1.53
Sulfonylurea	235 (21.36%)	14,168 (35.49%)	< 0.001	38.35%	35.69%	0.06
DPP-4i	124 (11.27%)	17,038 (42.68%)	< 0.001	50.59%	42.66%	0.16
SGLT2i	1100 (100%)	0 (0%)	< 0.001	100%	13.48%	3.58
TZD	102 (9.27%)	2705 (6.78%)	0.002	5.35%	7.03%	0.07
Glinides	28 (2.55%)	960 (2.40%)	0.764	1.49%	2.49%	0.07
Acarbose	90 (8.18%)	3011 (7.54%)	0.429	5.60%	7.83%	0.09
GLP1-RA	12 (1.09%)	352 (0.88%)	0.415	0.87%	0.91%	0.00
Insulin	135 (12.27%)	6748 (16.90%)	< 0.001	21.41%	17.43%	0.10
Lab (baseline)						
HbA1c, %	8.1 ± 1.8	8.3 ± 2.2	0.008	8.1 ± 11.6	8.3 ± 2.2	0.02
Hemoglobin	13.4 ± 2.3	12.5 ± 2.4	< 0.001	13.1 ± 17.5	12.5 ± 2.4	0.05
Hematocrit	43.7 ± 5.6	41.8 ± 5.9	0.438	43.2 ± 18	41.6 ± 6	0.12
Creatinine	0.9 ± 0.5	0.8 ± 0.3	< 0.001	1 ± 3.2	0.8 ± 0.3	0.09
eGFR	91.5 ± 32.5	97.5 ± 33.5	< 0.001	86.5 ± 228	97.4 ± 34	0.07
AST	34.7 ± 38.3	37.0 ± 82.4	0.123	33.2 ± 224	37.1 ± 84.9	0.02
ALT	37.2 ± 35.7	37.2 ± 51.1	0.995	36.7 ± 245	37.2 ± 52.2	0.00
BNP	587.6 ± 1012.6	367.9 ± 670.5	< 0.001	210.2 ± 3493.2	373.9 ± 690.1	0.07
NT-pro BNP	2163.3 ± 3,132.5	2567.8 ± 5,903.5	0.698	2094 ± 10,884.3	2524.2 ± 5977.9	0.05
Follow-up (years)	0.8 ± 0.8	1.5 ± 1.15	< 0.001	1.6 ± 7.2	1.5 ± 1.2	0.02

*ACEi* angiotensin converting enzyme inhibitor, *ALT* alanine transaminase, *ARB* angiotensin receptor blockers, *ARNI* angiotensin receptor-neprilysin inhibitor, *ASCVD* atherosclerotic cardiovascular disease, *AST* aspartate transaminase, *BNP* brain natriuretic peptide, *CCB* calcium channel blockers, *DM* diabetes mellitus, *DPP-4i* dipeptidyl peptidase-4 inhibitor, *eGFR* estimated glomerular filtration rate, *GLP1-RA* glucagon-like peptide 1 receptor agonist, *HbA1c* hemoglobin A1c, *LVEF* left ventricular ejection fraction, *NT-proBNP* N terminal pro B type natriuretic peptide, *OHA* other hypoglycemic agent, *SGLT2i* sodium glucose co-transporters 2 inhibitor, *TZD* thiazolidinedione

Outcomes of primary interest included heart failure hospitalization, acute coronary syndrome (including ST-elevation myocardial infarction, non-ST-elevation myocardial infarction, and unstable angina), ischemic stroke, and all-cause mortality. All-cause mortality was defined by withdrawal from the national health insurance (NHI) [18]. In Taiwan, since all citizens were abided by the law to be insured in the NHI program, a withdrawal from NHI is equivalent to the expiration (death) of this citizen. Each patient was followed until the day of outcome occurrence, date of death or December 31, 2019, whichever came first.

### Statistical analysis

To reduce the potential confounding when comparing outcomes between the study groups (SGLT2i as first-line treatment vs. metformin as first-line treatment), we used the inverse probability of treatment weighting (IPTW) method based on the propensity scores. The propensity score was estimated using a multivariable logistic regression model in which the study group was regressed on the selected covariates listed in Table 1 where the follow-up month was replaced with the index date. IPTW generates a synthetic population in which treatment assignment is independent of measured baseline covariates and therefore allows us to estimate average treatment effect similar to a randomized controlled trial. We used stabilized weight to mitigate the impact of extreme value of estimated propensity score. The balance of covariates between the groups before and after IPTW was checked using the absolute value of standardized difference (STD) between the groups, where a value less than 0.1 was considered negligible difference and a value ranged 0.1–0.2 was considered small difference. Risk of death from any cause between groups was compared using a Cox proportional hazard model. Competing risk regression (CRR) was performed with heart failure hospitalization, myocardial infarction, and cerebrovascular accident A *p* value < 0.05 was considered to be statistically significant. No adjustment of multiple testing (multiplicity) was made in this study. All statistical analyses were performed using commercial software (SAS 9.4, SAS Institute, Cary, NC, USA).

## Results

### Study population

**Fig. 1:**
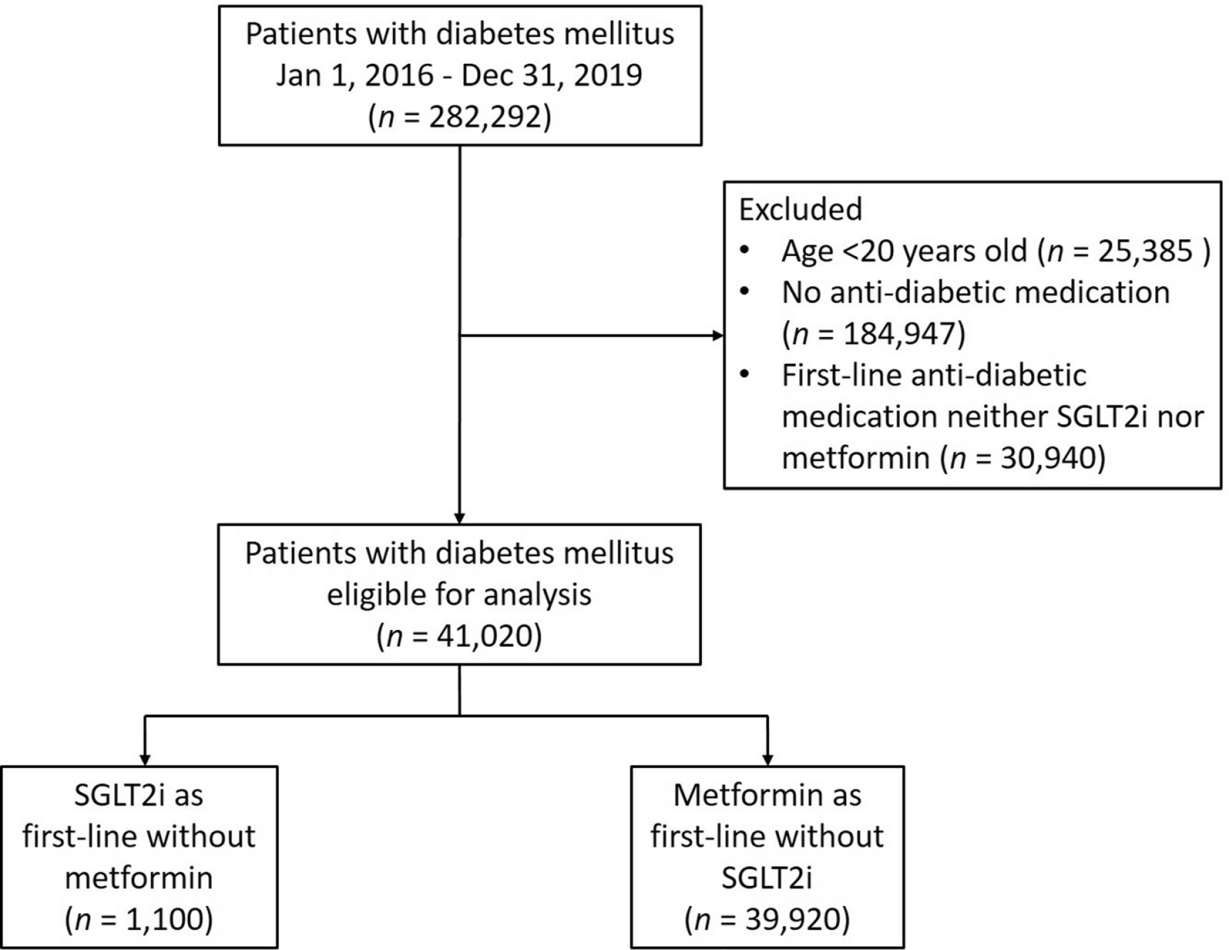
Study design and screening criteria flow chart for the inclusion of T2DM patients with SGLT2i as first-line treatment and metformin as first-line treatment

There were 282,292 patients with T2DM identified between January 1, 2016 and December 31, 2019 identified in the CGRD. After exclusion criteria, a total of 41,020 patients were enrolled, and separated into 1100 patients with SGLT2i as first-line treatment and 39,920 patients with metformin as first-line treatment (Fig. 1). Using IPTW, almost all variables such as age, sex, diabetes duration, LVEF, medication, selected lab results, and follow-up period have ASMD < 0.1 (Table 1). The mean age in SGLT2i as first-line treatment group was 57.6 ± 13.0 with 63.36% male and the mean age in metformin as first-line treatment group was 59.3 ± 12.9 with 56.03% male. The mean follow-up diabetes duration was 1.6 ± 7.2 years and 1.5 ± 1.21 years for SGLT2i and metformin as first-line treatment patients respectively. When the patient return to clinic after 3 months, those patients with A1c goal was not achieved, second anti-diabetic medication was added (Table 1).

### Cardiovascular events and all-cause mortality during follow up

**Fig. 2:**
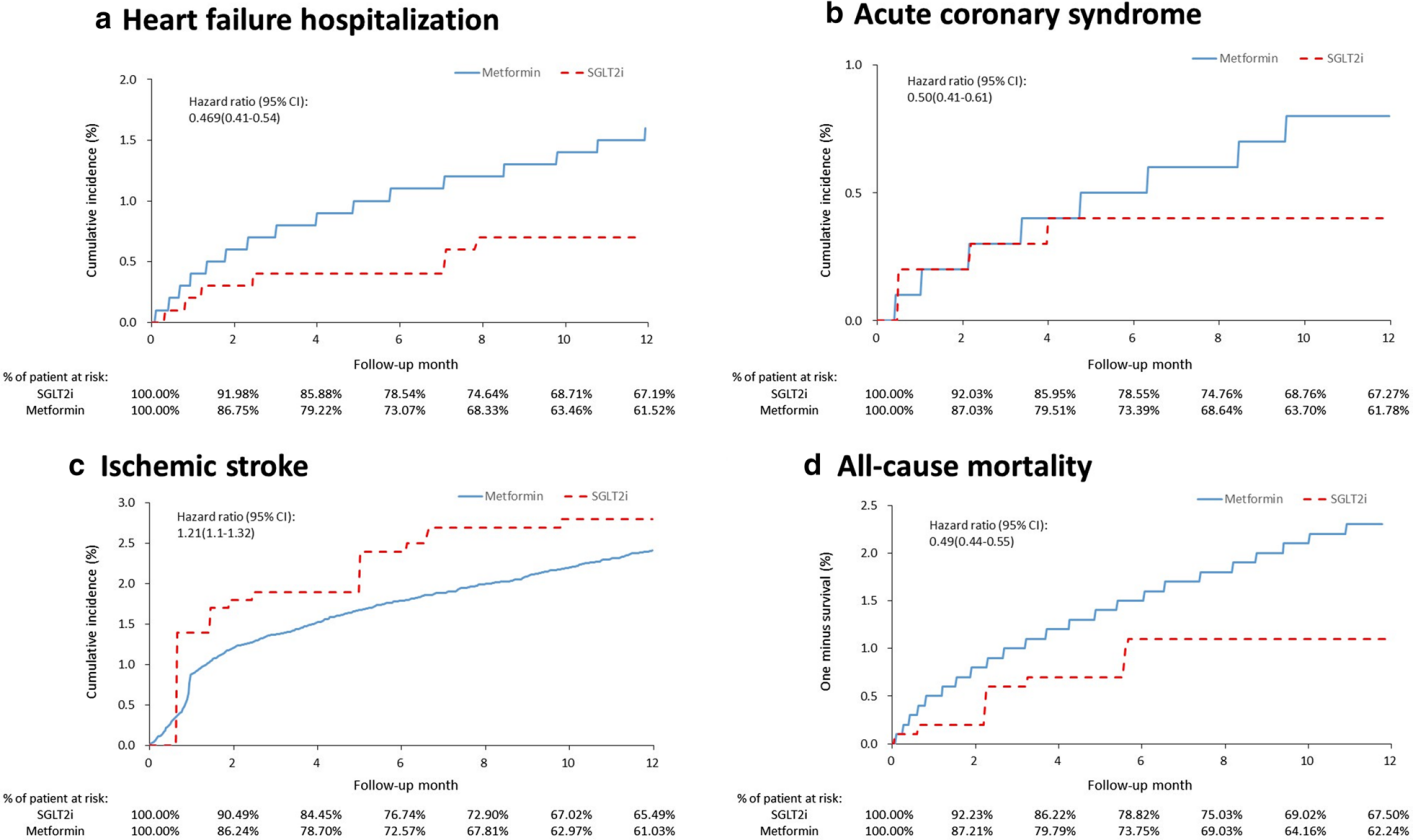
Cumulative incidence in heart failure hospitalization (**a**), acute coronary syndrome (**b**), ischemic stroke (**c**), and one sinus survival in all-cause mortality (**d**) in T2DM patients prescribed with either SGLT2i or metformin as first-line treatment

As shown in Table 2, patients with SGLT2i as first-line treatment had lower risks compared to patients with metformin as first-line treatment at 1-year outcomes in HF hospitalization (0.63% vs 1.26%), acute coronary syndrome (0.35% vs 0.66%), ischemic stroke (2.53% vs 2.01%), and all-cause mortality (1.05% vs. 1.95%). In addition, during one year follow-up, the hazard ratio (HR) of patients on SGLT2i as first-line treatment to patients on metformin as first-line treatment were HR 0.47 (95% CI 0.41–0.54, p < 0.0001) in heart failure hospitalization, HR 0.50 (95% CI 0.41–0.61, p < 0.0001) in acute coronary syndrome, HR 1.21 (95% CI 1.10–1.32, p < 0.0001) in ischemic stroke, and HR 0.49 (95% CI 0.44–0.55, p < 0.0001) in all-cause mortality (Fig. 2a–d).

**Table 2:** Primary outcomes at 1-year follow-up

	SGLT2i (*n* = 1100) (%)	Metformin (*n* = 39,920) (%)	Hazard ratio (95% CI)	*p *value
Heart failure hospitalization	0.63	1.26	0.47 (0.41–0.54)	< 0.0001
Acute coronary syndrome	0.35	0.66	0.50 (0.41–0.61)	< 0.0001
Ischemic stroke	2.53	2.01	1.21 (1.10–1.32)	< 0.0001
All-cause mortality	1.05	1.95	0.49 (0.44–0.55)	< 0.0001

## Discussion

This is the first study to compare the outcomes of T2DM patients in patients prescribed with SGLT2i or metformin as first-line treatment. Our study showed decreased events in heart failure hospitalization, acute coronary syndrome, and all-cause mortality when patients prescribed with SGLT2i as first-line treatment. However, there was increased events of ischemic stroke in patients prescribed with SGLT2i compared to metformin as first-line treatment.

### Previous studies

Patients with T2DM frequently have multiple coexisting conditions, in particular hypertension, hyperlipidemia, and cardiovascular disease (CVD) are the most prevalent [19]. Studies showed that that CVD was a comorbidity in approximately 1/3 patients with T2DM, with 29.1% having atherosclerosis, 21.2% coronary heart disease, 14.9% HF, 14.6% angina, 10.0% myocardial infarction, and 7.6% had experienced stroke [20, 21]. Previously, metformin rather sulfonylurea or insulin had been shown benefits in T2DM patients in the United Kingdom Prospective Diabetes Study (UKPDS) in decreased risks of macrovascular complications and all-cause mortality that persisted after trial was over [22, 23]. Therefore, metformin is still considered as the first-line pharmacological therapy in the latest 2020 ADA guidelines.

The rationales by which SGLT2 is provide the CV benefits that have been observed in recent studies remain to be elucidated. In the context of SGLT2 inhibition, there are multiple mechanisms that may contribute to the observed findings such as: SGLT2i-induced BP lowering may be associated with glycosuria and consequent negative energy balance, natriuresis and weight loss [24, 25]. The resultant reduction in weight and visceral fat deposition may contribute to decreased stiffness [26]. SGLT2i has also been linked to a reduction in epicardial fat, a biologically highly-active tissue involved in leptin and the renin–angiotensin–aldosterone system (RAAS) signaling, and may thereby be cardioprotective [27]. Moreover, SGLT2 inhibition may improve cardiac metabolism and bioenergetics by elevating the production of ketones, allowing myocardial cells requiring less oxygen to metabolize, and thus improving myocardial oxygen efficiency [28]. Thus, synergistic modulation of these factors via SGLT2 inhibition is proposed to play a significant role in the reduction of risk for the development of CVD.

It has been shown that empagliflozin significantly ameliorated myocardial oxidative stress injury and cardiac fibrosis in diabetic mice [29] and ipragliflozin increased adipocyte size associated with decreased expression of pro-inflammatory and fibrosis-related genes in abdominal perivascular adipose tissue of Western-type-fed mice [30]. The overexpressed SGLT1 in cardiomyocytes may represent a potential pharmacological target for cardioprotection [31]. Beneficial effects of SGLT2i on LV diastolic functional parameters for T2DM patients have also been described for both dapagliflozin [32] and canagliflozin [33]. Moreover, SGLT-2i reduced hospitalization for heart failure compared with DPP-4i [34] and canagliflozin was associated with a lower risk of heart failure admission to hospital and with a similar risk of MI or stroke in direct comparisons with three different classes of non-gliflozin drugs [35]. Finally, it has been debated that the results of EMPA-REG OUTCOME can be applied to patients with T2DM with a broader CV risk profile, including people at low risk of CVD [36].

### Current study

The fact that a large proportion of people with T2DM who are managed in routine practice have concomitant CVD raises the important question of whether it is effective to treat patients by guideline suggested metformin as first-line treatment or newer classes of anti-diabetic medication were not studied. All in all, CVD is associated with significant morbidity and mortality in people living with T2DM, and may account for nearly half (50.3%) of deaths in this population. This is markedly higher than the global mortality rate for CVD of 31% [37].

In this study, we reported the findings of decreased events in heart failure hospitalization, acute coronary syndrome, and all-cause mortality at 1-year follow-up in patients with T2DM prescribed with SGLT2i rather than metformin as first-line treatment. Although such results may be hinted by the new class of anti-diabetic medication in the landmark trials, SGLT2i has not been studied in this context with metformin. We found SGLT2i has a nearly equal risk reductions across heart failure hospitalization, acute coronary syndrome, and all-cause mortality with HR of 0.47–0.50, compared with metformin when prescribed to patients as first-line treatment under appropriate clinical discretion.

On the other hand, we also found that there was an increased events in ischemic stroke in the patients given with SGLT2i compared to metformin as first-line treatment group. A possible reason could be relating to the increased hypovolemia and hypotension caused by SGLT2i that may contribute to the increased incidence of ischemic stroke similar to the rationales underlying increased incidences of ischemic limbs in the CANVAS study. In addition, in the EMPA-REG OUTCOME study, there was a nonsignificant increase in the risk of stroke (HR 1.18; 95% CI 0.89–1.56), and in the CANVAS Program, there was a nonsignificant decrease in the risk of stroke (HR, 0.87; 95% CI 0.69–1.09) [38]. A meta-analysis of 42 trials with a total of 61,076 patients with type 2 diabetes showed that the risk of ischemic stroke was not reduced after SGLT2 inhibitor treatment in patients with type 2 diabetes (OR = 0.95, 95% CI 0.85–1.07, p = 0.42) [39]. Since clinical trials have shown good evidences that SGLT2i have cardiovascular benefits, and the role of SGLT2i was thus upgraded in the updates to the guidelines for diabetes treatment. Our study confirmed that is reasonable to prescribe SGLT2i vs metformin as first-line treatment regimen. At the same time however, our study also cautioned on the possible negative effect of SGLT2i on ischemic stroke, and clinicians must make appropriate judgement when prescribing these medications.

In summary, until recently there is no clear evidence that SGLT2i should replace metformin as first-line treatment in T2DM patients unless there is compelling evidence. Our study showed that it is reasonable practice for physicians to consider SGLT2i as first-line treatment in the context to reduce incidence of heart failure hospitalization, acute coronary syndrome and all-cause mortality in these patients.

### Limitations

There are several limitations in epidemiologic data from NHIRD. First, the study enrolled patients with diabetes mellitus being followed up at major teaching hospitals and tertiary-care medical centers, therefore the atherosclerotic burden could be higher than total diabetic population in Taiwan with selection bias. Second, using ICD-9-CM codes for patient screening and enrollment may miss some cases for which conditions were coded incorrectly. Third, detailed examination reports were not available in this claim-based database, therefore the exact extent and clinical development of ischemic stroke could not analyzed. Forth, due to small number of patients that SGLT2i could be prescribed as monotherapy, there was not enough information to directly compare the outcome in patients prescribed with SGLT2i versus metformin as monotherapy. Last, since our study consisted of nearly homogenous racial background, application of the results to other populations requires further studies.

## Conclusion

In patients with T2DM, SGLT2i as first-line treatment may be associated with decreased events of heart failure hospitalization, acute coronary syndrome, and all-cause mortality, compared with metformin as first-line treatment. However, there may be an increased events of ischemic stroke using SGLT2i compared to metformin.

## Data Availability

All data will be available upon reasonable request.
